# Shattering the Attentional Window: What Really Determines Capture by Abrupt Onsets and Color Singletons?

**DOI:** 10.5334/joc.269

**Published:** 2023-07-06

**Authors:** Mei-Ching Lien, Eric Ruthruff

**Affiliations:** 1 School of Psychological Science, Oregon State University, Corvallis, OR 97331, US; 2 Department of Psychology, University of New Mexico, Albuquerque, NM 87131, US

**Keywords:** Attention capture, target search mode, singleton detection, feature search, attentional window

## Abstract

There is emerging evidence that suppressing distractors occur to prevent capture by those distractors. Theeuwes (2022) claimed that the absence of capture is not because of suppression but rather because a difficult, serial search causes salient distractors to fall outside of the attentional window. Here, we question this attentional window view by describing evidence that (a) for color singletons, capture fails to occur with an easy search, and (b) for abrupt onsets, capture does occur in a difficult search. We argue that the critical factor determining capture by salient distractors is not the attentional window or search difficulty but rather target search mode (singleton vs. nonsingleton).

Theeuwes (2022) argued that salient distractors generally have the inherent power to capture attention against our will, at least when they are more salient than the target. To explain why salient distractors sometimes fail to produce capture effects, Theeuwes proposed that capture can be avoided when people engage in a difficult, serial search, yielding a small attentional window. The theory seems unlikely as, even in a difficult search, one still presumably first searches broadly to decide where to best begin the search. More importantly, there is a simple alternative explanation for when capture occurs.

The studies cited by Theeuwes (2022) as demonstrating capture by color singletons all involved targets that are themselves singletons (or “near singletons”). When the target is a singleton, participants tend to search for singletons (*singleton detection mode*), making the color singleton task-relevant (e.g., Bacon & Egeth, 1994; Lamy & Egeth, 2003; Ruthruff et al., 2020). When the target is not a singleton, participants instead search for a specific target feature (*feature search mode*). Search mode (singleton vs. feature) provides an elegant and compelling explanation of the existing data, making it unnecessary to invoke attentional windows (see also Kerzel & Huynh Cong, 2022).

To illustrate our point, Table 1 shows four possible categories of search, defined by whether search is easy/parallel or difficult/serial and whether the target is a singleton or not. The attentional window account predicts that the critical factor determining capture is search ease, whereas the target search mode account predicts that the critical factor is whether the target is a singleton. Both accounts predict capture in the upper left quadrant (easy search with singleton targets) and both predict no capture in the lower right quadrant (difficult search with nonsingleton targets). The studies described by Theeuwes (2022) all fall into the two quadrants for which both accounts agree. What is missing from his review are studies in the other two cells, for which the accounts actually disagree. Below we review these findings.

**Table 1:** Capture studies can be divided into four categories based on whether the search is easy (parallel) vs. difficult (serial) and whether the target is a singleton or a nonsingleton. According to the attentional window account, capture occurs with an easy, parallel search but not with a difficult, serial search. The target search mode account, however, predicts that capture occurs with a singleton target but not with a nonsingleton target. The shaded cells are the only ones where the two models make conflicting predictions. Yes: capture; No: No capture.


	EASY/PARALLEL SEARCH	DIFFICULT/SERIAL SEARCH

Singleton Target Search	Target Search Mode: Yes Attentional Window: Yes	Target Search Mode: **Yes** Attentional Window: **No**

Nonsingleton Target Search	Target Search Mode: **No** Attentional Window: **Yes**	Target Search Mode: No Attentional Window: No


## Easy Search with a Nonsingleton Target

**Figure 1:**
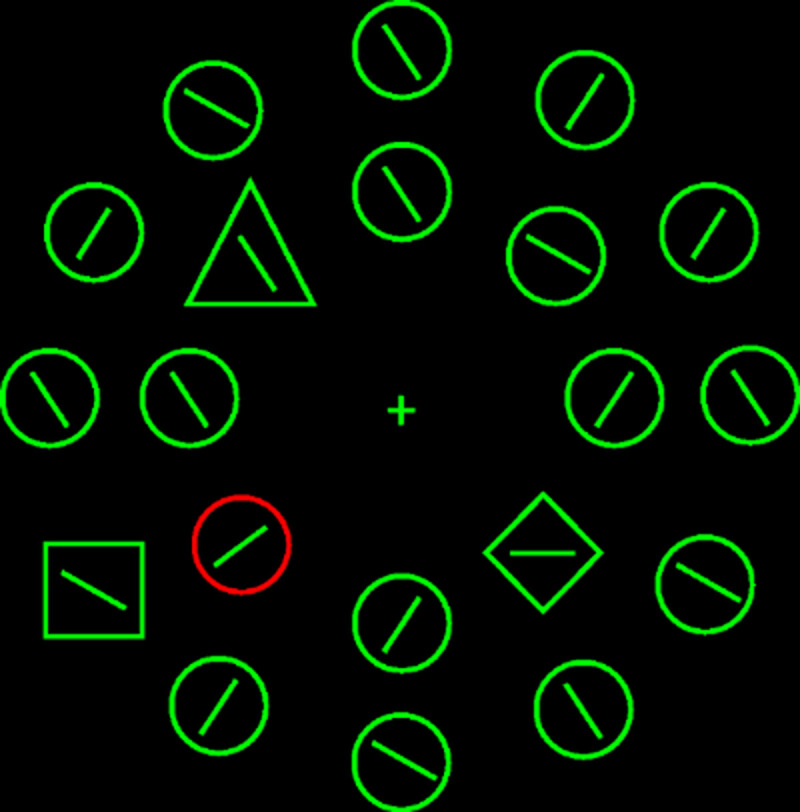
An example of the search display (setsize 20) in Theeuwes (2004).

Under easy search with a nonsingleton target, the attentional window account predicts capture but the target search mode account predicts no capture. Unfortunately, such studies seem rare. Theeuwes (2022) cited Theeuwes (2004; see Figure 1 for an example display) that reported capture in this cell (but see Wienrich and Jancyz, 2011, who failed to replicate the findings). As the original Theeuwes (2004) paper states, “observers searched… for a shape singleton (a diamond among circles)” (p. 67). But because there were two distractor singletons (a square and a triangle) in addition to the target singleton (a diamond), Theeuwes (2022) argued that “feature search mode had to be used”. However, this does not imply the absence of singleton detection mode. Searching for a singleton would help eliminate 17 of 20 search elements, reducing the effective setsize down to only 3 items.

**Figure 2:**
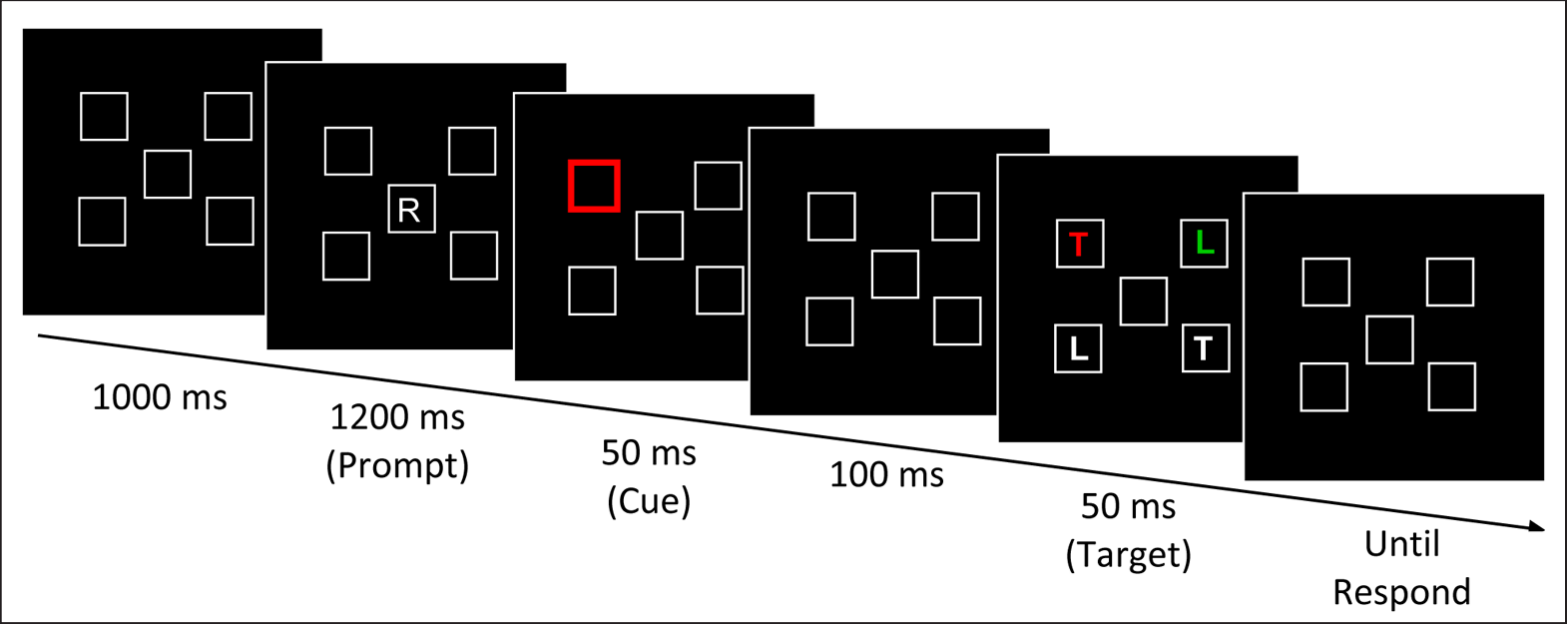
An example event sequence for the valid condition in Lien et al. (2010).

To test between the two competing accounts, what is needed is an easy search with a target that is clearly a nonsingleton. One such example is provided by Lien et al. (2010). Their displays had one letter in the target color (which changed from trial to trial), one in a distractor color, and two white letters (see Figure 2). Although their search was easy (a search slope of only about 6 ms/item), a color singleton precue produced little capture effect. A critic could reasonably note that the setsize was only four and it is logically possible that participants rapidly disengaged from the precue, before the target arrived. But note that when Folk et al. (1992; Experiment 4) used these same conditions – 4-item displays with precuing – combined with a singleton target, they observed large capture effects (40+ ms cue validity effect).

## Difficult Search with a Singleton Target

Under difficult search with a singleton target, the target search mode account predicts capture but the attentional window account predicts no capture. Studies meeting this requirement are also relatively rare. However, one potential example is Ruthruff et al. (2020; Experiments 1 and 2) where participants searched for a circle (a singleton target) amongst a homogeneous background of ovals. In some conditions the distractor ovals were nearly circular, creating a very difficult search; mean response time (RT) was 1,041 ms (Experiment 1), nearly double that of the easy search (610 ms). Nevertheless, the color singletons produced unusually large cue validity effects (121 ms in Experiment 1 and 70 ms in Experiment 2). Clearly, difficult search does not eliminate capture effects, as suggested by the attentional window account. In Experiment 3, making the search display distractors heterogenous (discouraging singleton search mode) virtually eliminated cue validity effects (1 ms). Search RT was nearly unchanged. Thus, the dramatic decrease of the cue validity effect cannot be explained by search difficulty, but it can easily be explained by target search mode.

Capture under difficult search is even more clear for the case of abrupt onsets. In fact, Gaspelin et al. (2016) found that increasing search difficulty reliably increased capture effects by abrupt onsets (see also Gaspelin et al., 2012). Their most difficult visual search produced an enormous capture effect of 141 ms (see also Ruthruff et al., 2020, Experiment 4). This search is much more difficult, with longer RTs, than the visual searches that Theeuwes (2022) claims to have stifled capture by color singletons.

## Concluding Remarks

The view that capture is determined by salience has been questioned by numerous findings of no capture by color singletons. Theeuwes (2022) proposed that this is because the search was too difficult, promoting serial search and a small attentional window that excludes salient distractors. We have reviewed evidence that search difficulty is not the key factor. For abrupt onsets, capture occurs even with very difficult searches. For color singletons, capture does not occur even for the easiest of searches, unless the color singleton is made task relevant by promoting singleton detection mode. The literature on capture by salient color singletons is predominated by studies promoting singleton detection mode and we believe that is unfortunate.

## References

[1] Bacon, W. F., & Egeth, H. E. (1994). Overriding stimulus-driven attentional capture. Perception & Psychophysics, 55, 485–496. DOI: 10.3758/BF03205306 8008550

[2] Folk, C. L., Remington, R. W., & Johnston, J. C. (1992). Involuntary covert orienting is contingent on attentional control settings. Journal of Experimental Psychology: Human Perception and Performance, 18(4), 1030–1044. DOI: 10.1037/0096-1523.18.4.1030 1431742

[3] Gaspelin, N., Ruthruff, E., & Lien, M.-C. (2016). The problem of latent attentional capture: Easy visual search conceals capture by task-irrelevant abrupt onsets. Journal of Experimental Psychology: Human Perception and Performance, 42, 1104–1120. DOI: 10.1037/xhp0000214 26854530 PMC4977216

[4] Gaspelin, N., Ruthruff, E., Lien, M.-C., & Jung, K. (2012). Breaking through the attentional window: Capture by abrupt onsets versus color singletons. Attention, Perception, and Psychophysics, 74, 1461–1474. DOI: 10.3758/s13414-012-0343-7 22806409

[5] Kerzel, D., & Huynh Cong, S. (2022). Search mode, not the attentional window, determines the magnitude of attentional capture. Attention, Perception, and Psychophysics. DOI: 10.3758/s13414-022-02582-4 PMC10806210 36207666

[6] Lamy, D., & Egeth, H. E. (2003). Attentional capture in singleton-detection and feature-search modes. Journal of Experimental Psychology: Human Perception and Performance, 29(5), 1003–1020. DOI: 10.1037/0096-1523.29.5.1003 14585019

[7] Lien, M.-C., Ruthruff, E., & Johnston, J. C. (2010). Attention capture with rapidly changing attentional control settings. Journal of Experimental Psychology: Human Perception and Performance, 36, 1–16. DOI: 10.1037/a0015875 20121291

[8] Ruthruff, E., Faulks, M., Maxwell, J. W., & Gaspelin, N. (2020). Attentional dwelling and capture by color singletons. Attention, Perception, and Psychophysics, 82, 3048–3064. DOI: 10.3758/s13414-020-02054-7 32483661

[9] Theeuwes, J. (2004). Top-down search strategies cannot override attentional capture. Psychonomic Bulletin & Review, 11(1), 65–70. DOI: 10.3758/BF03206462 15116988

[10] Theeuwes, J. (2022). The attentional capture debate: When can we avoid salient distractors and when not? Journal of Cognition. DOI: 10.5334/joc.251 PMC10327859 37426061

[11] Wienrich, C., & Janczyk, M. (2011). Absence of attentional capture in parallel search is possible: a failure to replicate attentional capture in a non-singleton target search task. Attention, Perception & Psychophysics, 73(7), 2044–2052. DOI: 10.3758/s13414-011-0183-x 21805210

